# Neuroinflammation in Alzheimer’s disease-associated sensory dysfunction: mechanistic links, actionable targets and therapeutic strategies

**DOI:** 10.3389/fnagi.2026.1838634

**Published:** 2026-07-06

**Authors:** Yanjiao Xu, Guimei Zhang, Xinran Cui, Li Sun

**Affiliations:** Department of Neurology, First Hospital of Jilin University, Jilin University, Changchun, Xinmin, China

**Keywords:** Alzheimer’s disease, neuroinflammation, olfaction, retina, visual impairment

## Abstract

Alzheimer’s disease (AD) is the most common cause of dementia and major public-health challenge in aging societies worldwide. Accumulating evidence suggests that olfactory and visual deficits can precede overt cognitive symptoms and are closely associated with amyloid-β deposition, pathological tau phosphorylation, and disease progression. Early sensory abnormalities in AD likely arise from converging pathological processes. Among these, chronic neuroinflammation marked by microglial and astrocytic reactivity, inflammasome activation and increased pro-inflammatory mediators might play a pivotal role linking sensory-circuit injury to neurodegeneration. A coherent synthesis of the inflammatory mechanisms underlying early olfactory and visual impairment in AD remains limited, and putative molecular pathways and interventions have not been fully integrated. We aimed to identify AD-related olfactory and visual or retinal abnormalities, combine core inflammatory pathways and their interactions with amyloid-β and tau pathology, and summarize actionable targets and candidate interventions along a “receptor–intracellular signaling-inflammasome-effector” axis, to inform earlier-stage detection and mechanism-guided intervention in AD.

## 1 Introduction

The World Health Organization (WHO) has estimated that 55.2 million people were living with dementia in 2019, and that this will reach 139 million by 2050 (World Health Organization, 2021). Alzheimer’s disease (AD) is the most common dementia subtype. It is neuropathologically characterized by amyloid-β (Aβ)-associated neurotoxicity and hyperphosphorylated tau pathology (Ising and Heneka, 2018). Neuroinflammation has been repeatedly implicated as an active driver of AD pathogenesis and progression (Shal et al., 2018). Neuroinflammation, Aβ deposition, and aberrant tau phosphorylation enhance each other. Sustained inflammation can accelerate Aβ aggregation and tau pathology, whereas accumulating Aβ and phosphorylated tau further perpetuate inflammatory activation (Ising et al., 2019). Microglial and astrocytic activation, inflammasome activation, and the release of pro-inflammatory cytokines contribute to chronic neuroinflammation in AD. These processes are closely associated with oxidative stress and neuronal degeneration or apoptosis, and further promote the recruitment of inflammatory cells and the propagation of inflammatory cascades, ultimately resulting in neuronal loss (Thakur et al., 2023).

Sensory visual, olfactory, and hearing deficits can emerge early in AD and often persist throughout the disease course (Woodward et al., 2017; Koronyo et al., 2023; Li et al., 2025). Given its early occurrence, sensory dysfunction may have important value as an early warning indicator of AD, and investigating the underlying pathophysiological mechanisms may provide important insights into disease development and potential therapeutic strategies. Neuroinflammation is a major contributor to sensory dysfunction in AD. Glial activation and elevated pro-inflammatory mediator production in the olfactory and visual systems are closely linked to olfactory and visual deficits in AD (Gaire et al., 2024; Yu et al., 2024). Neuroinflammation may reduce odor responsiveness through olfactory neuronal degeneration and apoptosis (Hasegawa-Ishii et al., 2020; Song et al., 2025), and is closely associated with retinal dysfunction resulting from neurovascular unit disruption, photoreceptor degeneration, and neuronal loss (Moriuchi et al., 2020; Ramirez et al., 2017; Mohamed et al., 2015; Ashok et al., 2020). These alterations may aggravate the neuropathological burden and cognitive impairment in AD. However, a coherent synthesis of the inflammatory mechanisms underlying early olfactory and visual impairment in AD remains limited, and putative molecular pathways and intervention points have not been fully integrated. Unlike previous reviews focusing on the clinical features of sensory dysfunction, sensory cortical pathology, or individual sensory modalities, this review provides neuroinflammation-centered synthesis of the mechanisms underlying AD-related olfactory and visual/retinal abnormalities. We integrate pathological changes across both central sensory pathways and peripheral sensory structures, including the retina, within a unified framework. Beyond summarizing the reciprocal interactions between neuroinflammation and Aβ/tau pathology, we systematically map potential therapeutic targets and intervention strategies spanning receptors, intracellular signaling pathways, inflammasomes, and downstream effectors. By connecting sensory dysfunction with neuroinflammatory mechanisms and therapeutic intervention, this review highlights potential avenues for early detection and target identification in AD.

## 2 Subsections

### 2.1 Neuroinflammation in AD-related olfactory dysfunction

Olfactory dysfunction typically manifests as reduced odor detection, impaired odor discrimination, and weakened odor memory (Dan et al., 2023). Accumulating evidence indicates that olfactory dysfunction emerges in the early stages of AD, precedes overt cognitive impairment, and is associated with disease progression (Woodward et al., 2017; Roberts et al., 2016). Notably, a selective decline in odor discrimination is considered among the earliest detectable behavioral changes and might serve as a candidate early marker of AD (Dan et al., 2023).

The olfactory epithelium (OE), bulb (OB), (OT) tract and cortical olfactory region coexist with Aβ and tau pathology during inflammatory changes in persons with AD (Yu et al., 2024; Arnold et al., 2010; Bathini et al., 2019). These processes appear to supplement each other, reducing neuronal activity and disrupting olfactory signal transmission, while aggravating circuit dysfunction by promoting oxidative stress and neurodegeneration (Ising et al., 2019; Bathini et al., 2019). Astrocytes and microglia are obviously activated in the OBs of triple-transgenic mouse models of Alzheimer’s disease with hyposmia. This is reflected by increased glial fibrillary acidic protein (GFAP), ionized calcium-binding adaptor molecule 1 (Iba1) and elevated transcription of pro-inflammatory interleukin (IL)-6 and tumor necrosis factor-α (TNF-α; Yu et al., 2024). The locus coeruleus (LC), the principal source of noradrenaline (NA) for olfactory processing, undergoes early axonal degeneration in AD mice, resulting in reduced noradrenergic input to the OB and concomitant olfactory deficits. Meyer et al. (2025) identified microglia-mediated axonal phagocytosis as a key driver of this process and uncovered a novel LC–OB–microglia–axon pathway linking neuroinflammation to olfactory dysfunction in AD. Furthermore, prosaposin promotes astrocyte proliferation and a pro-inflammatory phenotype. Elevated prosaposin signaling in the entorhinal cortex (EC) was accompanied upregulated reactive astrocytes in the human hippocampus entorhinal system (Roberts et al., 2016). This suggested that an inflammatory astrocytic program contributes to early injury in olfaction-related cortical regions.

Receptors in the OE detect odorants under physiological conditions, then conveys them *via* olfactory sensory neurons (OSNs) to the OB. Mitral/tufted cells (M/Ts) then integrate the input and relay signals *via* the OT to higher-order regions such as the EC to generate olfactory perception. Damage at any step of this pathway can compromise olfactory function. As a key relay hub, structural and functional abnormalities of the OB are closely linked to hyposmia. Dysfunction and OSN loss can drive OB atrophy and are considered important contributors to olfactory impairment. Amyloid-β and phosphorylated tau accumulation is frequently accompanied by inflammatory pathology in the OBs of patients with AD, and these changes correlate with the severity of olfactory impairment (Yu et al., 2024). Neuroinflammatory changes in the OB such as glial activation and release of inflammatory cytokines such as TNF-α can reduce OSN excitability and are associated with reduced cellularity and OB atrophy (Hasegawa-Ishii et al., 2020; Yeh et al., 2023). Excessive microglial activation promotes synaptic loss in OSNs and degeneration of LC axons, thereby contributing to olfactory dysfunction, including impaired odor responsiveness and discrimination (Meller and Greer, 2025; Meyer et al., 2025). Heightened astrocytic reactivity might inhibit M/T cell activity, increase odor-detection thresholds, and weaken discrimination ability (Ung et al., 2020). Moreover, pro-inflammatory mediators released by activated glia, such as IL-6, might induce apoptosis and OSN necrosis, further compromising olfactory transmission (Song et al., 2025). Collectively, these findings suggest that neuroinflammation contributes to the onset and progression of olfactory dysfunction in AD by promoting OSN synaptic loss, cell death, and maladaptive circuit remodeling.

### 2.2 Neuroinflammation in AD-related visual dysfunction

Accumulation of Aβ plaques and phosphorylated tau in the lens, retina and visual cortex of patients with AD is associated with complex visual abnormalities (Gaire et al., 2024). Visual impairment, a common comorbidity of AD, often manifests as reduced color and contrast sensitivity or visual field defects, and might be related to retinal Aβ deposition (Gaire et al., 2024). Damage to the visual cortex and visual pathways can further disrupt higher-order visual processing, resulting in visuospatial agnosia, environmental disorientation, and prosopagnosia (Cerquera-Jaramillo et al., 2018). Retinal dysfunction has been the most extensively investigated among these changes. The retina shares a common embryological origin with the central nervous system. It exhibits similar cellular composition, glial responses, and barrier structures, making it a potential window into AD-related brain pathology. Accumulating evidence indicates that retinal amyloid deposition, tau pathology, and inflammatory changes follow trajectories that parallel those in the brain during different stages of AD (Koronyo et al., 2023). Therefore, we summarized evidence of neuroinflammatory changes to the AD retina and discuss their mechanistic relevance and potential as therapeutic targets.

Amyloid-β deposition and phosphorylated tau accumulation have been detected in the retina of patients with AD and mouse models (Habiba et al., 2021, Koronyo et al., 2017, Gaire et al., 2024), which correlate with cerebral Aβ burden, tau pathology, and the degree of brain atrophy (Koronyo et al., 2023). However, Williams et al. found no evidence of phosphorylated tau pathology in post-mortem retinal tissues from 19 patients with AD (Williams et al., 2017). Such inconsistencies may reflect differences in sample size, disease stage, or detection approaches across studies. Accumulating Aβ and phosphorylated tau induce inflammatory responses, activates glial cells, and triggers the release of cytokines and other mediators that promote apoptosis in retinal and cerebral tissues (Liu et al., 2014).

Retinal glial populations mainly comprise microglia and macroglia, namely astrocytes and Müller cells (Reichenbach and Bringmann, 2020). Accumulating evidence links AD-related retinal pathology to excessive activation of glial cells. Activated astrocytes are increased in post-mortem retinas from patients with AD (Gaire et al., 2024). These are accompanied by obvious microglia activation, elevated osteopontin expression (Grimaldi et al., 2019) and enhanced Iba1 immunoreactivity (Xu et al., 2022). MicroRNA-155 is considered a key regulator that drives the transition of microglia from a homeostatic (M0) state to various disease-associated microglia/microglial neurodegenerative phenotypes. MicroRNA-155 plays important roles in inflammatory and immune responses. Disease-associated microglia are widely distributed around Aβ plaques in the retinas of mice with AD. Conditional ablation of microRNA-155 in these cells attenuates retinal inflammation and reduces IL-2, IL-5, IL-12, IL-6, IL-10, and interferon-gamma (IFN-γ) expression while preserving the inner blood retinal barrier and limiting vascular amyloid pathology, which alleviates AD-related retinal damage (Shi et al., 2022). Exposure to Aβ induces Müller cell gliosis and a pro-inflammatory phenotype in animal models (Dinet et al., 2012). In contrast, the immunoreactivity of Müller cell markers GFAP and glutamine synthetase is reduced in post-mortem human retinas, possibly reflecting a loss of Müller cells after prolonged exposure to Aβ and related pathology (Xu et al., 2022). Mechanistically, excessive glial activation promotes tau phosphorylation and neurofibrillary tangle formation *via* p38 mitogen-activated protein kinase (MAPK), and activates nuclear factor kappa-light-chain-enhancer of activated B cells (NF-κB) signaling to drive glutamate excitotoxicity and impair synaptic plasticity, ultimately leading to widespread neuronal loss and axonal degeneration (Thakur et al., 2023). Glial activation can further reduce retinal blood flow by suppressing nitric oxide synthase (NOS) activity, thus aggravating retinal pathology and impairing Aβ clearance (Thakur et al., 2023). Excessive Aβ deposition upregulates complement C3 expression in surrounding neuronal synapses and axons, which promotes microglial phagocytosis and leads to the loss of retinal ganglion cells and rod axons, retinal thinning, and photoreceptor degeneration, ultimately exacerbating visual impairment (Moriuchi et al., 2020; Ramirez et al., 2017). Chronic inflammation can also downregulate retinal iron-transport proteins, increase intracellular iron levels and oxidative stress (Ashok et al., 2020), and promote retinal pathology by disrupting the neurovascular unit (Mohamed et al., 2015).

Inflammatory responses mediated by activation of the NLR family pyrin domain containing 3 (NLRP3) inflammasome and complement pathways have been implicated in AD-related visual dysfunction. The expression of IL-1 beta (IL-1β) and complement C3 is increased in post-mortem retinas of humans with AD and mouse models (Grimaldi et al., 2019). A single intravitreal injection of Aβ into mouse retinas activates NF-κB signaling, which promotes NLRP3 inflammasome activation and IL-1β and TNF-α release (Lei et al., 2017). Aβ, IL-1α, and TNF-α can further induce complement C3 expression and consequently accelerate synaptic deterioration in AD (Gupta et al., 2021; Grimaldi et al., 2019).

Collectively, excessive glial activation and elevated pro-inflammatory mediators in retinas associated with AD highlight a central role of neuroinflammation in visual dysfunction and support the potential of retinal inflammatory signatures as biomarkers of disease progression (Doustar et al., 2020).

### 2.3 Shared pathways, potential therapeutic targets, and intervention strategies

Sustained inflammation might undermine key endogenous protective mechanisms against neuronal injury in AD. Olfactory and visual dysfunctions in AD are closely associated with neuroinflammatory processes, and auditory impairment might likewise exacerbate cognitive decline through inflammation-related mechanisms (Ren et al., 2024). Notably, apolipoprotein E4 (APOE4) carrier status is also associated with susceptibility to AD-related retinal and olfactory dysfunction. Glial inflammatory responses such as modulated NF-κB related pathways reshaped by APOE4 influence the vulnerability of sensory systems (Arnaud et al., 2022). Knock-in mouse models carrying APOE4 have retinal structural abnormalities and visual impairment, with neuroinflammation likely acting as an important factor (Abhyankar et al., 2025). Neuroinflammation serves as a central hub linking cognitive and sensory impairment in AD by inducing oxidative stress, glutamate excitotoxicity, synaptic loss, neuronal degeneration, and apoptosis. Lost OSNs and bulb neurons with reduced odor responsiveness, leads to OB atrophy and impaired olfactory function (Hasegawa-Ishii et al., 2020; Song et al., 2025). Disruption of the neurovascular unit, reduced blood flow, photoreceptor degeneration, and retinal ganglion cell loss collectively contribute to retinal dysfunction (Moriuchi et al., 2020; Ramirez et al., 2017; Mohamed et al., 2015; Ashok et al., 2020). Therefore, delineating neuroinflammatory mechanisms underlying sensory dysfunction in AD is essential for identifying therapeutic targets and optimizing intervention strategies.

### 2.4 Inflammatory mechanisms underlying sensory dysfunction in AD

**Figure 1:**
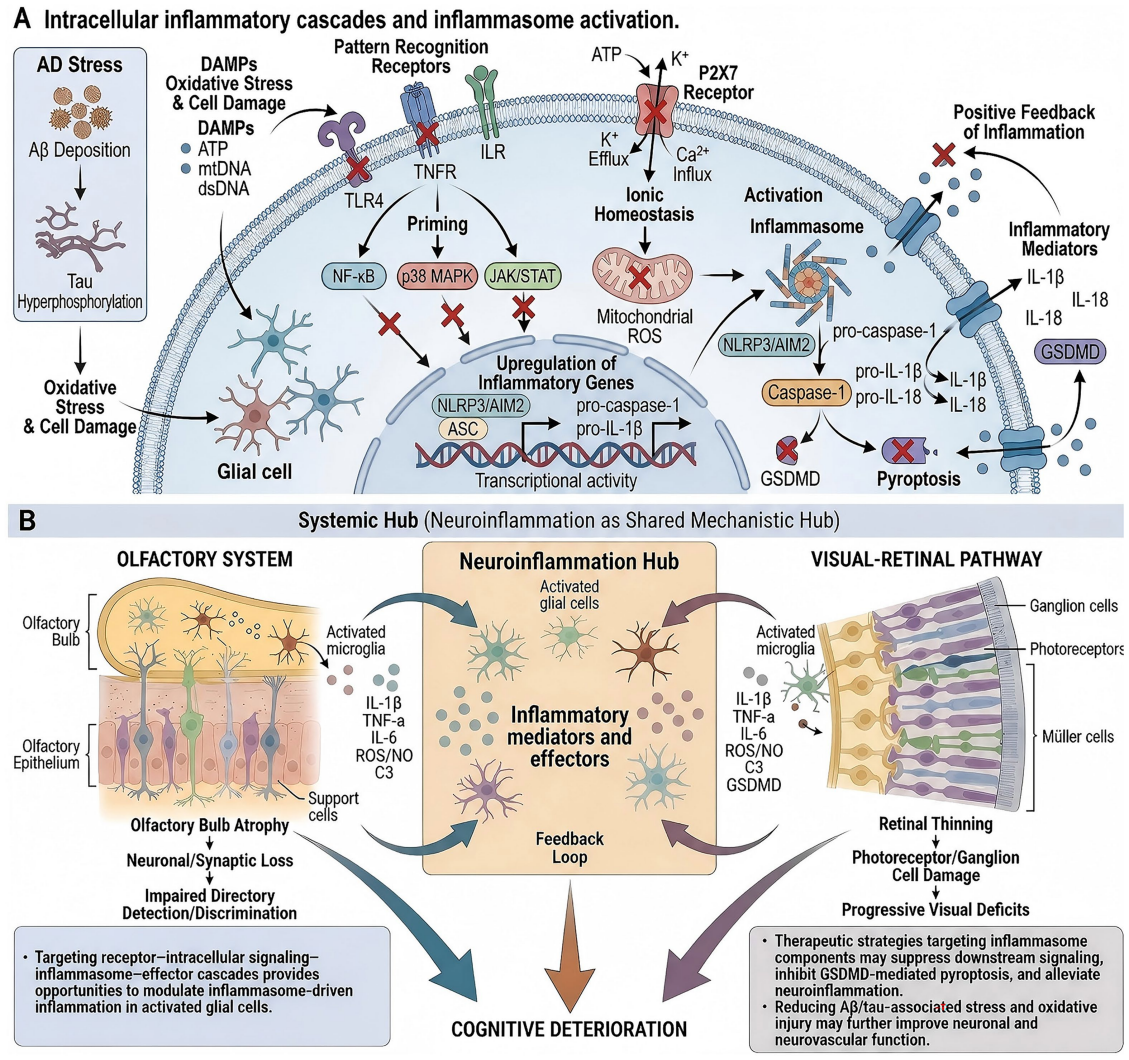
Neuroinflammation may represent a shared mechanistic hub linking sensory dysfunction and cognitive decline in Alzheimer’s disease. **(A)** AD-related stress, amyloid-β (Aβ) accumulation, and tau hyperphosphorylation are associated with oxidative damage and DAMP release. These signals can activate pattern-recognition receptors and initiate inflammasome priming through the NF-κB, MAPK, and JAK/STAT pathways, leading to increased expression of inflammasome-related components. Ionic imbalance and mitochondrial reactive oxygen species (ROS) further promote NLRP3 or AIM2 inflammasome assembly, caspase-1 activation, IL-1β and IL-18 maturation, and gasdermin D–mediated pyroptosis, thereby amplifying inflammatory signaling. **(B)** Chronic activation of microglia, astrocytes, and Müller cells in the olfactory and visual-retinal systems promotes sustained release of pro-inflammatory mediators, contributes to synaptic dysfunction and impaired neuronal survival, and is associated with sensory dysfunction. Neuroinflammation may therefore serve as a convergent hub linking sensory impairment and cognitive decline in AD and represents a potential therapeutic target.

Activated glial cells in the OB and retina elevate pro-inflammatory cytokine levels with direct neurotoxic effects and promotes further Aβ deposition. Pathological accumulation of Aβ and phosphorylated tau induces mitochondrial dysfunction and oxidative stress, resulting in neuronal injury and the release of damage-associated molecular patterns (DAMPs), such as mitochondrial DNA (mtDNA). These DAMPs subsequently activate innate immune signaling pathways and further amplify neuroinflammatory responses. Excess Aβ itself can act as a DAMP to activate glial cells. Extracellular Aβ in the OBs and retinas of patients with AD can bind pattern-recognition receptors such as toll-like receptors (TLRs) to activate signaling cascades that are dependent on NF-κB. In contrast, intracellular Aβ directly promotes the assembly of NLRP3 inflammasomes and the expression of inflammatory mediators (Nakanishi et al., 2018). Activated caspase-1 promotes maturation and release of pro-inflammatory IL-1β and IL-18, and cleaves gasdermin D (GSDMD), which triggers pyroptotic cell death (He et al., 2015). Pyroptosis exposes intracellular danger signals, further amplifying NLRP3 inflammasome activation. Double-stranded DNA can activate absent in melanoma 2 (AIM2) inflammasomes and further aggravate inflammatory responses by activating apoptosis-associated speck-like protein containing a CARD (ASC)-dependent caspase-1. Concurrently activated adenosine triphosphate (ATP)-gated purinergic receptor P2X7, ligand-gated ion channel on glial cells, induces K^+^ efflux and Ca^2+^ influx, disrupting cellular and mitochondrial ion homeostasis and promoting mitochondrial reactive oxygen species release, which further facilitates inflammasome activation (Yao et al., 2024; Figure 1).

### 2.5 Therapeutic targets and potential intervention strategies

For a more systematic and transparent synthesis of therapeutic targets, this section was developed based on a predefined literature search strategy and evidence classification framework. English-language articles published between January 2000 and December 2025 were retrieved from PubMed, Embase, and Web of Science Core Collection. A combination of controlled vocabulary and free-text terms was used, including “Alzheimer’s disease,” “amyloid-β,” “tau,” “neuroinflammation,” “microglia,” “astrocyte,” “inflammasome,” “NLRP3,” “NF-κB,” “JAK/STAT,” “TREM2,” “olfactory dysfunction,” “olfactory impairment,” “retinal dysfunction,” “visual impairment,” “sensory dysfunction,” “therapeutic target,” “intervention,” and “treatment.” Search terms were adapted as appropriate for each database. Studies were included if they: (1) involved patients with AD, AD animal models, or AD-related cellular models; (2) investigated neuroinflammatory mechanisms, inflammatory signaling pathways, potential therapeutic targets, or intervention strategies; and (3) reported outcomes related to neuroinflammation, AD pathology, cognition, or sensory function. Duplicate publications, editorials, commentaries, conference abstracts, and studies unrelated to the topic were excluded. Additional relevant studies were identified through manual screening of reference lists from eligible articles and related reviews. Because evidence directly targeting olfactory or visual dysfunction in AD remains limited, studies demonstrating modulation of neuroinflammation and improvement of AD-related pathology or cognitive outcomes, but without direct assessment of sensory function, were included as indirect evidence. In addition, interventions validated in other neurodegenerative disorders, retinal diseases, or inflammation-related models and targeting pathways discussed in this review were included as putative evidence. Based on the study population and the relevance of outcomes to sensory dysfunction, the available evidence was categorized into three levels: direct evidence, indirect evidence, and putative evidence (Table 1).

**Table 1:** Compounds that improve AD sensory disorder *via* neuroinflammation regulation.

Compounds	Level of evidence	Dose	Route	Duration	Sex	Experimental model	Action targets	Inflammatory assessment parameters	Sensory endpoints	Concurrent cognitive assessment	Cognitive outcome improvement	References
Masitinib	A	60 mg/kg/d	p.o.	1 wk	Male	Acrolein-induced sporadic AD mouse model (C57BL/6 mice)	CSF1R (inhibition), the NF-κB/NLRP3/caspase-1 pathway (inhibition)	Iba1, NF-κB, NLRP3, caspase-1, IL-1β	Odor detection	Yes	Spatial memory and learning	Jia et al. (2025)
TO90	A	50 mg/kg/d	i.g.	1 wk. (3 d before and 4 d after injection)	/	Aβ intravitreal injection C57BL/6J mouse model	The NF-κB/NLRP3 pathway (inhibition)	Iba1, NLRP3, caspase-1, TNF-α, IL-6, IL-1β	Scotopic adaptation function	No	/	Lei et al. (2017)
Raddeanin A	A	10 mg/kg/d	p.o.	9 wk	Female and male	3 × Tg-AD mice	The NF-κB/NLRP3/caspase-1 pathway (inhibition), the Wnt/β-catenin pathway (inhibition)	NLRP3, caspase-1, ASC, IL-1β, IL-18	Retinal cell apoptosis and BRB damage	No	/	Wang et al. (2024)
Salvianolic acid B	A	20 mg/kg/d	i.g.	3 mo	Female and male	5 × FAD mice	NLRP3 inflammasome (inhibition), β-site amyloid precursor protein cleaving enzyme 1 (BACE1) (inhibit its function and promote its degradation)	Iba1, NLRP3, IL-10, TNF-α, IL6, IL-1β	Scotopic/photopic adaptation, and photopic negative response	No	/	Wang et al. (2023)
Vinpocetine	A	15 mg/kg/d	i.p.	3 d	Female	Long-Evans rats with intraocular Aβ injection	The NF-κB/NLRP3/caspase-1 pathway (inhibition)	NF-kB, IL-1β, IL-18, TNF-α, NLRP3, caspase-1	RPE cell inflammation level	No	/	Liu et al. (2014)
Docosahexaenoic acid (DHA)	B	DHA diet	p.o.	Euthanasia at 3/6/12 mo (2 wk. post-weaning)	/	APOE4 transgenic C57BL/6 mice	NF-κB, caspase (inhibition)	Iba1, caspase	Odor detection, discrimination, habituation	Yes	Olfactory recognition memory	González et al. (2023)
Nicotinamide adenine dinucleotide (NAD)	B	Water containing 12 mM NAD analog nicotinamide riboside (NR)	p.o.	8 mo	Female and male	C57BL/6J mice	NAD (supplementation)	GFAP, Iba1	Odor detection	No	/	Dan et al. (2023)
PLX5622	B	1,200 ppm PLX5622-formulated chow	p.o.	10–24 wk	/	5xFAD mice	CSF1R (inhibition)	microglia	/	No	Aβ burden	Spangenberg et al. (2019)
Berbamine (BBM)	B	25 and 50 mg/kg/d	p.o.	3 wk	male	APP/PS1 mice	the rapamycin-binding domain of mTOR	Iba1, TNF-α, IL-1β	/	Yes	Spatial memory and learning	Ge et al. (2025)
TAK-242	B	2 mg/kg/d	i.p.	28 d	male	APP/PS1 mice	TLR4 (inhibition)	CD11b + microglia, NF-κB、NLRP3	/	Yes	Spatial memory and learning	Cui et al. (2020)
Hesperetin	B	50 mg/kg/d	i.p.	6 wk	Male	Aβ_1-42_ + C57BL/6 mice	TLR4/NF-κB signaling (inhibition)	GFAP, Iba1, TLR4, NF-κB	/	Yes	Short-term memory	Ikram et al. (2019)
Atorvastatin	B	5 and 10 mg/kg/d	p.o.	4 wk. (3 wk. pre-injection, 6 d post-injection)	male	Sprague–Dawley rats injected Aβ_1–42_	the TLR4/TRAF6/NF-κB pathway (inhibition)	GFAP, Iba1, TLR4, TRAF6, NF-κB	/	Yes	Spatial memory and learning	Wang et al. (2018)
DA4-JC	B	0.1, 1, and 10 nmol/kg/d	i.p.	8 wk	Female and male	APP/PS1 mice	GLP-1/GIP receptor (Agonist)	TNF-α, IL-1β	/	Yes	Spatial memory	Maskery et al. (2020)
Artemisinin	B	1, 5 and 10 mg/kg/d	i.p.	30 d	Male	3xTg AD mice	The IRE1/NF-κB signaling pathway (inhibition)	IRE1, IKK, NF-κB, GFAP, TNF-α, IL-6	/	Yes	Spatial memory and learning	Chen et al. (2025a,b)
Salidroside (SAL)	B	3 g/kg/d	i.g.	12 wk	Male	Diabetic rat model	The PI3K/Akt/GSK-3β/NF-κB pathway	NF-κB, TNF-α, IL-6, IL-1β	Retinal Müller cell inflammation & RGC count	No	/	Feng et al. (2024)
TfRMAb-TNFR	B	3 mg/kg	i.p.	12 wk., 3 d/wk	female	3xTg-AD mice	TNF-α receptor	Iba1, TREM2	/	No	Hippocampal Aβ degradation signaling	Jagadeesan et al. (2024)
VX-765	B	50 mg/kg, 3 times/wk	i.p.	12 times	Female and male	APPS^w/Ind^ transgenic mouse, C57BL/6 J WT mice	Caspase-1 (inhibition)	Iba1, GFAP, IL-10, IL-1β, TNF-α	/	Yes	Spatial and episodic memory	Flores et al. (2022)
AL002c	C	30 mg/kg	i.p.	once	/	CV-KO-5XFAD mice and R47H-KO-5XFAD mice	TREM2 (inhibition)	Iba1 + microglia and DAM microglia	/	No	/	Wang et al. (2020a,b)
Curcumin	C	10, 30, and 50 μM	/	1 h	/	Murine BV2 microglia cell	The signaling pathway of JAK/STAT/SOCS (inhibition)	IL-6, IL-1β, TNF-α	/	/	/	Porro et al. (2019)
Calycosin	C	5, 10, and 20 μM	/	24 h	/	Primary rat spinal astrocytes	The GP130/JAK/STAT pathway	p-JAK2, p-STAT3, p-AKT, GP130, IL-6	/	/	/	Song et al. (2022)
Metformin	C	200 mg/kg/d	i.p.	14d	/	C57BL/6 J mice	Nrf2 (inhibition)	Nrf2, NF-κB, IL-4, IL-10, TNF-α	/	No	/	Akter et al. (2024)
Pomiferin	C	1 μM	/	12 h	/	BV2 cells	The Akt/Nrf2 pathway (agonist), the NF-κB pathway (inhibition)	IL-6, TNF-α, iNOS, COX2, NF-κB	/	/	/	Zhao et al. (2022)
Bupropion	C	40, 80 and 160 mg/kg/d	p.o.	5 d	Male	Wistar rats	The TLR4/NF-κB pathway (inhibition)	TLR4, NF-κB, TNF-α	/	No	/	Rashidian et al. (2020)
Edaravone dexborneol	C	1.5 mg/kg, twice per day	i.v.	7, or14d	Male	C57/BL6 mice	NF-κB signaling (inhibition)	Reactive astrocytes	/	No	/	Chen et al. (2024)
Icariside II	C	5, 10, and 20 μM	/	1 h	/	Primary astrocytes extracted from neonatal rat brains	The IKK/IκB/NF-κB/BACE1 signaling pathway (inhibition)	NF-κB, IKK-α, IKK-β, IκB-α	/	No	Aβ generation	Zheng et al. (2020)
Photobiomodulation	C	810 nm diode laser (150 mW output power), 60 min/d	/	2 wk	male	Sprague–Dawley rats	Lcn2/JAK2-STAT3 (inhibition)	Iba1, GFAP, Lcn2, JAK2, pJAK2, STAT3, pSTAT3	/	No	/	Wang et al. (2021)
Sailuotong capsule	C	16.5 and 33 mg/kg	i.g.	28d	Male	Sprague–Dawley rats	Lcn2 (inhibition)	IL-6, IL-12, IL-1α, chemokine CXCL10, GFAP, LCN2, p-JAK2, p-STAT3	/	Yes	Spatial and memory	Zhang et al. (2019)
Iron chelators (deferoxamine and deferiprone)	C	0–150 μM deferoxamine, 0–500 μM deferiprone	/	0-72 h	/	Primary astrocytes extracted from WT and Lcn2 KO mice	Lcn2 (inhibition)	Astrocyte Lcn2 expression level	/	/	/	Dekens et al. (2020)
Tranilast	C	100 μM	/	24 h	/	Normal human keratinocytes (NHKs)	NLRP3 (inhibition)	NLRP3, caspase-1, IL-1β	/	/	/	Zhuang et al. (2020)
MCC950	C	5 μM	/	/	/	Primary human cells and CD14 + monocytes extracted from C57BL/6 and Asc^−/−^ mice	NLRP3 (inhibition)	NLRP3, caspase-1, IL-1β	/	/	/	Coll et al. (2019)
Sennoside A	C	1, 10, 30 mg/kg	i.p.	once	Male	C57/BL6 mice, murine macrophages and human monocytes	Caspase-1 (inhibition)	Caspase-1, IL-1β, IL-18	/	/	/	Wu et al. (2022)
Roxadustat (FG-4592)	C	10 mg/kg	i.p.	once	/	Balb/c mice	CD73, AIM2 inflammasome (inhibition)	CD73, AIM2	/	/	/	Yang et al. (2023)

(1) A, direct evidence, direct sensory Alzheimer’s disease evidence; B, indirect evidence, general Alzheimer’s disease evidence/sensory non-Alzheimer’s disease evidence; C, speculative evidence, non-Alzheimer’s disease inflammatory evidence. (2) “p.o.,” oral gavage; “i.p.,” intraperitoneal injection; “i.g.,” intragastric administration; “i.v.,” intravenous injection via tail vein. (3) “d,” day(s); “wk,” week(s); “mo,” month(s); “h,” hour(s). (4) CSF1R, colony stimulating factor 1 receptor; DHA, docosahexaenoic acid; NAD, nicotinamide adenine dinucleotide; NF-κB, nuclear factor kappa B; NLRP3, nucleotide-binding domain and leucine-rich repeat containing receptor family pyrin domain containing 3; AIM2, absent in melanoma 2.

Preclinical studies have found that neuroinflammation is a modifiable component of AD-associated sensory dysfunction that has therapeutic potential to ameliorate both cognitive deficits and sensory impairments. A bioactive compound isolated from sea anemones, Raddeanin A, salvianolic acid B, and the liver X receptor agonist T0901317, can ameliorate AD-related retinal dysfunction by suppressing NF-κB signaling and NLRP3-mediated retinal inflammation (Wang et al., 2024; Lei et al., 2017; Wang et al., 2023), and vinpocetine attenuates Aβ-induced retinal inflammation (Liu et al., 2014). Dietary docosahexaenoic acid and nicotinamide adenine dinucleotide supplementation reduce neuroinflammation and Aβ deposition in the OB of AD mice, which improves cognitive and olfactory performance (González et al., 2023; Dan et al., 2023; Lessard-Beaudoin et al., 2021). Several compounds and natural products, although not yet directly tested for sensory outcomes in AD, modulate distinct inflammatory pathways and might be potential therapeutic candidates for sensory dysfunction. Key intervention targets in several regulatory levels from glial activation to receptor signaling, intracellular pathways, inflammasomes, and downstream effectors, and highlight representative compounds targeting each level are summarized below.

#### 2.5.1 Targeting microglia

Microglia are resident innate immune cells of the central nervous system that undergo pronounced morphological and transcriptional changes in response to pathogens or tissue damage (Jurga et al., 2020). Microglial phenotypes have traditionally been classified as pro-inflammatory (M1) or anti-inflammatory (M2). However, accumulating evidence indicates that they adopt more complex transcriptional states such as disease-associated microglia or microglial neurodegenerative phenotypes in diseases such as AD. During the early phases of AD, microglia facilitate Aβ clearance through phagocytic and degradative mechanisms. Persistent pathological stimulation, however, drives chronic microglial activation, leading to excessive inflammasome signaling and the release of pro-inflammatory cytokines and chemokines. These processes amplify neuroinflammatory cascades and contribute to neuronal degeneration (Thakur et al., 2023). Experimental studies consistently show that limiting maladaptive microglial activation reduces neuroinflammation, promotes Aβ clearance, and ameliorates cognitive and sensory dysfunction (Han et al., 2022; Shi et al., 2025; Zhou et al., 2024; Jia et al., 2025).

Triggering receptor expressed on myeloid cells 2 (TREM2) and colony-stimulating factor 1 receptor (CSF1R) are abundantly expressed in microglia in humans with AD and animal models. Both TREM2 and CSF1R regulate microglia proliferation, migration, phagocytic capacity, and anti-inflammatory phenotype, thus influencing neuroinflammation and Aβ clearance (Han et al., 2022; Shi et al., 2025). Evidence suggests that TREM2-mediated microglial activation exerts protective effects during retinal photoreceptor degeneration (Zhou et al., 2024). Humanized agonistic anti-TREM2 monoclonal antibodies such as AL002 promote the expansion of anti-inflammatory microglia, enhance Aβ phagocytosis, and reduce neuroinflammation (Wang et al., 2020a). The neuroprotective effects of TREM2 might involve suppressing the phosphoinositide 3-kinase-protein kinase B-forkhead box O3a pathway and activation of Wnt/β-catenin signaling (Wang et al., 2020b; Liu et al., 2019; Zhu et al., 2025). Masitinib is an oral tyrosine kinase inhibitor with high selectivity for CSF1R that improves olfactory and cognitive deficits in mouse models of AD (Jia et al., 2025). Inhibitors of CSF1R such as pexidartinib (PLX3397) and PLX5622 reduce inflammation and promote Aβ plaque clearance by inducing microglial depletion or reactivity (Okojie et al., 2023; Nissen et al., 2018; Spangenberg et al., 2019). Emerging evidence indicates that neuroinflammatory responses and microglial functions exhibit substantial sex-dependent differences in AD (Fyfe, 2024; Reed and Keller-Norrell, 2023). Female and male microglia differ in transcriptional profiles, inflammatory reactivity, and responses to therapeutic interventions (Han et al., 2021). Notably, CSF1R inhibition by PLX3397 induces greater microglial depletion in male than in female mice, suggesting that the efficacy of microglia-targeted therapies may vary according to sex (Le et al., 2025). Despite these findings, sex-stratified analyses remain underrepresented in most preclinical and clinical studies. Future investigations should incorporate biological sex as a key variable to improve the translational relevance and precision of neuroinflammation-targeted therapies. Berbamine targets the FKBP12 rapamycin-binding domain of the mechanistic target of rapamycin complex in microglia (Ge et al., 2025), offering additional strategies to modulate microglial reactivity.

The immune checkpoint molecule T-cell immunoglobulin and mucin-domain containing 3, encoded by hepatitis A virus cellular receptor 2, is closely involved in maintaining microglial homeostasis in mouse models of AD. Genetic deletion of hepatitis A virus cellular receptor 2 promotes a neuroprotective microglial phenotype characterized by enhanced phagocytosis, reduced pro-inflammatory gene expression, and improved Aβ clearance and cognitive performance (Kimura et al., 2025). These findings suggest that microglial T-cell immunoglobulin and mucin-domain containing 3 is a potential therapeutic target for AD.

Beyond its central role in AD pathogenesis (Kanekiyo et al., 2014), *APOE4* may represent a potential target for microglial regulation. The transition of microglia from a homeostatic state to the disease-associated microglia phenotype is accompanied by marked transcriptional reprogramming, including *APOE* upregulation (Keren-Shaul et al., 2017). Evidence from cellular studies suggests that *APOE4* promotes DAM polarization in human AD and is positively associated with TREM2 expression (Lin et al., 2018). Moreover, *APOE4* carriers display elevated levels of TNF-α, IL-6, and IL-1β (Fan et al., 2017), supporting a role for *APOE4* in driving pro-inflammatory microglial responses.

#### 2.5.2 Targeting astrocytes

In response to Aβ, tau, and endogenous damage-associated signals, astrocytes acquire reactive phenotypes that amplify neuroinflammation, promote neurotoxicity, and contribute to pathological Aβ and tau accumulation. This shift amplifies inflammatory responses, exerts neurotoxic effects, and contributes to aberrant Aβ and tau accumulation (Gaire et al., 2024). Astrocytes in the retina, OB, and EC of persons with AD have prominent reactivity, which promotes glial scar formation and directly compromises sensory neurons by releasing neurotoxic mediators (Koronyo et al., 2023; Arnold et al., 2010). Numerous signaling pathways and pro-inflammatory cues tightly regulate astrocyte activation and polarization under chronic inflammatory conditions. Attenuating excessive astrocytic reactivity or interrupting signaling pathways that drive activation state might help to reduce the inflammatory burden within sensory pathways. Accordingly, targeting astrocyte reactivity has promise as a strategy to mitigate inflammatory amplification and preserve neuronal integrity in sensory pathways affected by AD (Ageeva et al., 2024; Lee et al., 2023).

#### 2.5.3 Targeting inflammatory receptors

The prototypical pattern-recognition receptor TLR4 is involved in innate immunity. It is expressed by microglia, astrocytes, and subsets of neurons, and upregulated in the peripheral and central tissues of persons with AD. This receptor recognizes Aβ and diverse DAMPs, which leads to the activation of NF-κB and MAPK signaling cascades that drive the excessive production of pro-inflammatory cytokines. This process amplifies glial reactivity and contributes to a neurotoxic microenvironment closely associated with Aβ accumulation and sustained neuroinflammation (Walter et al., 2007; Miron et al., 2018). The selective TLR4 inhibitor TAK-242 (resatorvid) promotes a shift of microglia from a pro-inflammatory, to an anti-inflammatory phenotype and suppresses NF-κB/NLRP3 signaling, which leads to attenuated neuroinflammation (Cui et al., 2020). Monophosphoryl lipid A is a detoxified derivative of lipopolysaccharide and a TLR4 agonist. It selectively upregulates scavenger receptor expression and enhances Aβ uptake, while inducing substantially weaker NF-κB activation than lipopolysaccharide, which avoids excessive inflammatory responses (Michaud et al., 2013; Kong and Ge, 2008). Hesperidin, ProBiotic-4, and atorvastatin alleviate neuroinflammation and improve learning and memory by suppressing the TLR4/NF-κB axis in animal models of AD (Ikram et al., 2019; Yang et al., 2020; Wang et al., 2018).

Metabolism-related receptors, including glucagon-like peptide-1 (GLP-1R), glucose-dependent insulinotropic polypeptide and glucagon receptors, have recently emerged as important modulators of neuroinflammatory processes. Agonists of GLP-1R such as GLP-1 and exendin-4, downregulate TLR4/NF-κB signaling and suppress MAPK pathways in models of AD. This reduces glial activation, lowers Aβ burden, and improves cognitive performance (Chen et al., 2018; Zhang et al., 2021). The neuroprotective effects of liraglutide might involve activation of the cyclic adenosine monophosphateprotein kinase A-cyclic adenosine monophosphate response elementbinding protein signaling pathway (Li et al., 2015). Dual GLP-1R/glucose-dependent insulinotropic polypeptide receptor and triple agonists have shown superior anti-inflammatory and cognitive benefits compared with individual GLP-1R agonists in amyloid precursor protein/presenilin 1 mouse models (Maskery et al., 2020; Salles et al., 2020; Tai et al., 2018). These findings suggest that coordinated modulation of several metabolic receptors offer a more effective strategy for controlling neuroinflammation.

#### 2.5.4 Targeting intracellular signaling pathways: Janus kinase-transducer and activator of transcription (JAK/STAT) and NF-κB

In response to extracellular stimuli, glial cells engage multiple intracellular signaling cascades such as NF-κB, MAPK, and JAK/STAT pathways that orchestrate reactive phenotypes and inflammatory gene expression. Among these pathways, NF-κB and signal of transducer and activator of transcription 3 (STAT3) are central transcriptional regulators that govern neuroinflammatory responses and glial reactivity (Ageeva et al., 2024). Accordingly, restraining aberrant activation of JAK/STAT and NF-κB signaling is a key strategy to attenuate glial activation and neuroinflammation.

The JAK/STAT pathway comprises membrane receptors, JAKs and STAT transcription factors that are tightly controlled by negative regulators such as suppressors of cytokine signaling, protein inhibitors of activated STATs and protein tyrosine phosphatases (Miklossy et al., 2013; Diop et al., 2022). Among these regulators, suppressors of cytokine signaling proteins play pivotal roles in constraining pathway activity by blocking STAT receptor interactions or promoting ubiquitin-mediated degradation of JAKs (Diop et al., 2022). Levels of activated or phosphorylated STAT3 are reduced in hippocampal neurons of patients with AD. Humanin and its derivative colivelin improve AD-related memory deficits *via* activation of STAT3 signaling (Chiba et al., 2009). Antisense oligonucleotides have also been explored as tools to modulate STAT3 expression and downstream inflammatory responses (Rusek et al., 2023). Curcumin suppresses JAK/STAT signaling by upregulating cytokine suppressors, which results in anti-inflammatory and neuroprotective effects (Porro et al., 2019). Isoflavonoids such as calycosin, as well as pharmacological JAK inhibitors including tofacitinib and baricitinib, also suppress JAK/STAT signaling and attenuate neuroinflammatory responses (Song et al., 2022; Rusek et al., 2023).

Nuclear factor-kappa B is a master regulator of inflammatory gene transcription and is persistently activated in reactive glial cells, where its activity correlates with AD progression and sensory dysfunction, including olfactory and visual impairment (Jong Huat et al., 2024; Lei et al., 2017; Palomino-Alonso et al., 2017). Activating NF-κB induces the transcription of inflammasome components, pro-inflammatory cytokines, and caspases. Activated NF-κB promotes complement C3 expression in astrocytes, which leads to dendritic disruption and impaired neuronal network integrity. Under basal conditions, NF-κB is sequestered in the cytoplasm by inhibitor of kappa B (IκB) proteins. Inflammatory stimuli trigger IκB phosphorylation and degradation that allows NF-κB to translocate to the nucleus and initiate transcription (Ageeva et al., 2024). O-GlcNAcylation mediated by O-GlcNAc transferase declines with aging in astrocytes and modulates inflammatory responses by regulating NF-κB signaling. Supplementation with GlcNAc attenuates inflammation and improves cognitive performance (Dong et al., 2023; Wheatley et al., 2019). Nuclear factor erythroid 2–related factor 2 (Nrf2) suppresses NF-κB signaling, reduces reactive astrocytosis and ameliorates cognitive deficits in models of AD (Nakano-Kobayashi et al., 2023). Metformin and pomiferin activate Nrf2 signaling, suppress NF-κB activity, reduce TNF-α and IL-1β production, and promote anti-inflammatory responses (Akter et al., 2024; Zhao et al., 2022). Bupropion, artemisinin, edaravone–dexborneol, icariside II, hydrogen sulfide, ent-sauchinone, and salidroside attenuate neuroinflammation by inhibiting NF-κB signaling, and some also reduce the Aβ burden (Rashidian et al., 2020; Chen et al., 2025b; Chen et al., 2024; Zheng et al., 2020; Zhao et al., 2023; Song et al., 2014; Feng et al., 2024). Masitinib improves olfactory and spatial learning deficits in AD models by modulating the NF-κB/NLRP3/caspase-1 axis (Jia et al., 2025).

Signaling hubs such as NF-κB and JAK/STAT are key regulatory nodes in the inflammatory networks underlying both cognitive and sensory dysfunction in AD, and therefore constitute promising therapeutic targets. These pathways also highlight astrocytes as critical cellular effectors, suggesting that astrocyte-directed interventions have substantial potential for alleviating sensory impairment in AD.

#### 2.5.5 Targeting pro-inflammatory mediators

Astrocyte reactivity is closely linked to sustained exposure to inflammatory mediators, including IL-1β, TNF-α, IFN-γ, IL-1α, and C1q (Lee et al., 2023). Consequently, inhibiting cytokine- and complement-driven astrocyte reactivity may represent a promising therapeutic strategy to attenuate the amplification of neuroinflammatory responses. Therapeutic modulation of the IL-1 signaling axis has been extensively explored in the context of neuroinflammatory disorders. Agents that target IL-1 such as the IL-1 trap rilonacept, the recombinant IL-1 receptor antagonist anakinra, and the anti–IL-1β monoclonal antibody canakinumab, exert anti-inflammatory effects by interrupting IL-1 signaling (Klein et al., 2021; Bami et al., 2020; Hosono et al., 2022). These agents have robust anti-inflammatory activity among inflammatory conditions and are becoming considered as potential modulators of neuroinflammatory responses. In the triple-transgenic mouse model of AD, biological TNF inhibitors upregulate microglial TREM2 expression and reduce mature Aβ plaque burden (Jagadeesan et al., 2024). These findings suggest that TNF-α signaling modulates Aβ clearance, at least in part, by shaping microglial phenotypic states.

Lipocalin-2 (Lcn-2) has recently attracted attention because of its strong association with inflammatory burden and progression from mild cognitive impairment to AD (Choi et al., 2011). It is secreted by reactive astrocytes and activated microglia, and promotes GFAP expression, NLRP3 activation and cytokine release under inflammatory conditions *via* NF-κB and JAK/STAT signaling pathways, while exacerbating neuronal injury by enhancing oxidative stress (Mondal et al., 2020; Tan et al., 2024; Xiang et al., 2022). Tau pathology amplifies the pro-inflammatory effects of Lcn-2, whereas neuronal Lcn-2 knockdown in models of AD restores synaptic plasticity and improves cognitive performance (Zhou et al., 2023). Several approaches have been explored to suppress Lcn-2 expression or activity. These include photobiomodulation, herbal formulations such as Sailuotong, and the iron chelators deferoxamine and deferiprone, which reduce Lcn-2 levels and conceivably attenuate neuroinflammation and cognitive deficits in experimental models (Wang et al., 2021; Zhang et al., 2019; Dekens et al., 2020). However, direct evidence linking Lcn-2 modulation to sensory dysfunction in AD remains limited. Moreover, whether Lcn-2 exerts exclusively pro-inflammatory effects in the nervous system remains controversial. For instance, deleting Lcn-2 does not significantly alter glial activation in J20 AD mice (Dekens et al., 2018). Such discrepancies likely reflect differences in disease stage, inflammatory milieu or experimental models, underscoring the need for systematic evaluation across disease contexts to clarify the role of Lcn-2 in neuroinflammation.

#### 2.5.6 Targeting inflammasome activation

**Figure 2:**
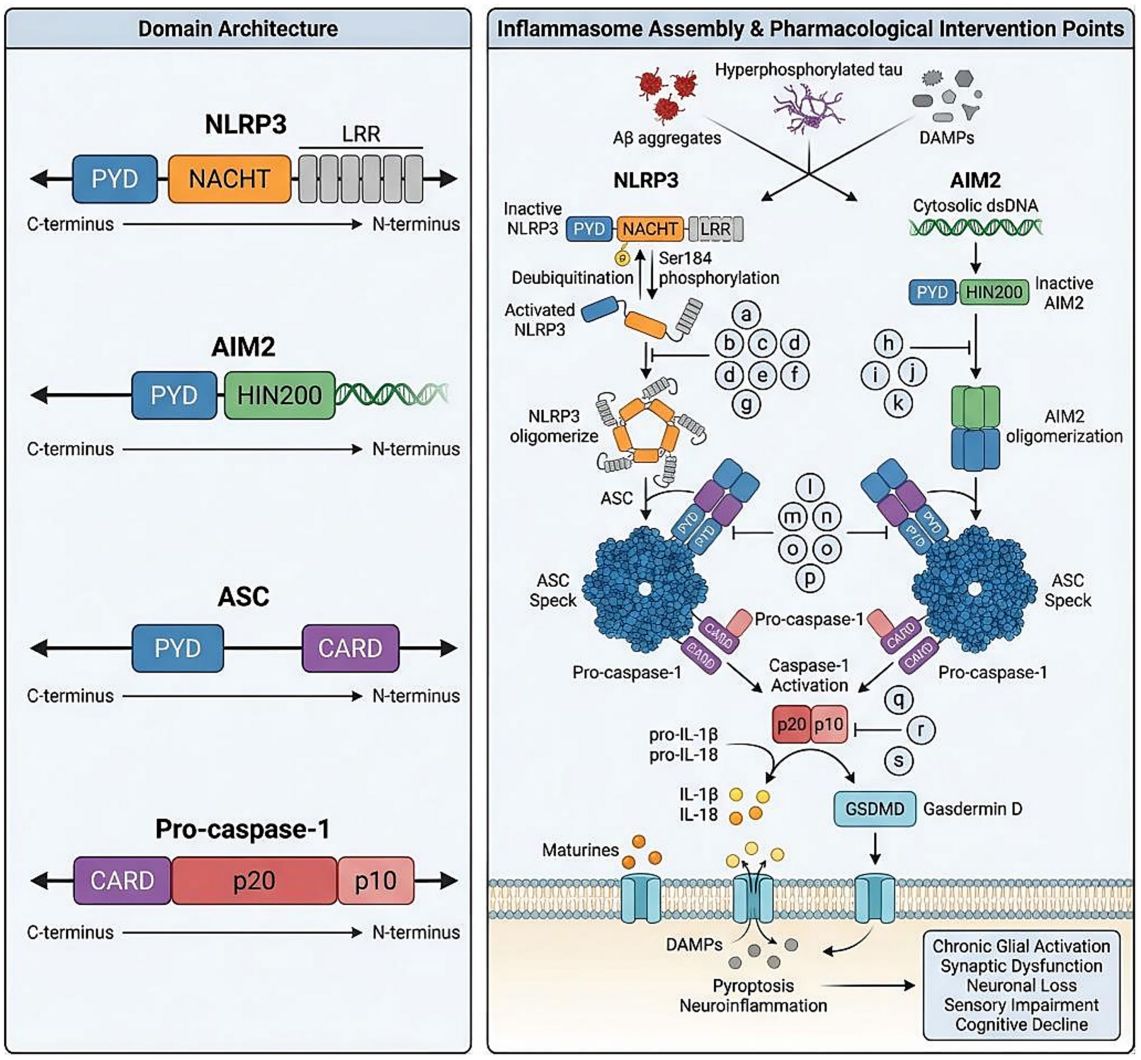
Inflammasomes as a shared molecular hub linking neuroinflammation to sensory dysfunction in AD. Stressors associated with AD, including amyloid-β, pathological tau, and DAMPs converge upon the activation of NLRP3 and AIM2 inflammasomes. Inflammasome assembly drives caspase-1–dependent maturation of IL-1β/IL-18 and gasdermin D–mediated pyroptosis, sustaining chronic glial activation and disrupting synaptic and neuronal integrity in olfactory and retinal pathways. Key structural domains, assembly steps and pharmacological intervention points are highlighted, underscoring neuroinflammation as a common mechanistic hub linking sensory deficits to cognitive decline. (a) MCC950; (b) tranilast; (c) CY-09; (d) oridornin; (e) tetrahydroquinoline; (f) OLT1177; (g) costunolide; (h) NIC-0102; (i) FG-4592; (j) EFLA-945; (k) POP3; (l) CAPE; (m) lonidamine; (n) brivilin A; (o) dehydrocostus; (p) obvatal; (q) pralnasasan; (r) VX-765; (s) sennoside A. AD, Alzheimer’s disease; AIM-2, absent in melanoma-2; DAMPS, damage-associated molecular patterns; OLT1177, dapansutrile.

Inflammasome activation represents a central amplification step in neuroinflammatory cascades and has emerged as a key mechanistic target. Inflammasomes consist of a sensor molecule, the adaptor ASC, and an effector protease, among which NLRP3 is the most extensively studied. Upon sensing DAMPs, NLRP3 undergoes conformational changes and oligomerization, recruits ASC *via* its pyrin domain, and subsequently activates caspase-1. Activated caspase-1 then drives IL-1β and IL-18 maturation and induces GSDMD cleavage, thereby triggering pyroptotic cell death (Yao et al., 2024). In AD, the NLRP3 inflammasome is widely regarded as a molecular hub linking Aβ accumulation to neuroinflammation and neuronal injury. Several small molecules inhibit NLRP3 inflammasome activation *via* direct or indirect mechanisms. For example, tranilast target the central nucleotide-binding and oligomerization domain NACHT to prevent ATP hydrolysis and NLRP3 oligomerization (Zhuang et al., 2020; Coll et al., 2019), whereas caffeic acid phenethyl ester and ZYIL1 primarily interfere with ASC recruitment and assembly (Yao et al., 2024; Yuan et al., 2023). Sennoside A attenuates inflammatory responses by inhibiting caspase-1 activity (Wu et al., 2022). VX-765 (belnacasan), a selective caspase-1 inhibitor that ameliorates neuroinflammation and cognitive deficits in animal models. However, potential hepatotoxicity associated with long-term administration remains a concern (Flores et al., 2022). NIC-0102 suppresses inflammasome activation by promoting NLRP3 ubiquitination and disrupting its interaction with ASC (Yao et al., 2024). Pyrin domain-only protein 3 inhibits AIM2 activation by competitively binding to its pyrin domain, whereas roxadustat (FG-4592) suppresses the AIM2 inflammasome assembly *via* a CD73-dependent mechanism (Khare et al., 2014; Yang et al., 2023). Non-steroidal anti-inflammatory drugs partially inhibit NLRP3 activation and IL-1β elevation, which alleviate neuronal injury (Yuan et al., 2023). Disulfiram can inhibit pore formation mediated by GSDMD, which is a key executor of pyroptosis (Yao et al., 2024). Collectively, inflammasome signaling, particularly *via* NLRP3 and AIM2 constitutes a major amplification node in AD-associated neuroinflammation. Pharmacological interference at different stages of inflammasome assembly and execution might a viable strategy to attenuate inflammatory damage and its downstream impact on sensory dysfunction (Figure 2).

#### 2.5.7 Targeting aberrant aβ aggregation

As the core pathological hallmarks of AD, Aβ deposition and tau hyperphosphorylation may impair visual and olfactory function through multiple mechanisms, including neuroinflammation and synaptic dysfunction. The olfactory bulb and visual cortex may represent vulnerable sites of early Aβ accumulation and may therefore be more likely to benefit from anti-Aβ therapy. Lecanemab, which targets soluble Aβ protofibrils (van Dyck et al., 2023), and donanemab, which targets pyroglutamate-modified Aβ (p3-Aβ) (Sims et al., 2023), have both been shown to reduce cerebral amyloid burden and slow disease progression, supporting the central role of Aβ in the AD pathological cascade. In the TRAILBLAZER-ALZ 2 trial, Donanemab achieved substantial amyloid clearance after 76 weeks of treatment and slowed cognitive and functional decline. Approximately 80% of participants with low or intermediate tau levels reached amyloid clearance at week 76 (Sims et al., 2023). However, the clinical application of anti-Aβ therapies remains limited by amyloid-related imaging abnormalities (ARIA). ARIA mainly includes cerebral edema or sulcal effusion (ARIA-E) and hemorrhagic manifestations, such as cerebral microhemorrhages and superficial siderosis (ARIA-H). *APOE4* genotype is one of the strongest risk factors for ARIA. The risk of ARIA increases with the number of *APOE4* alleles. *APOE4* homozygotes have a substantially higher risk of ARIA-E and ARIA-H than heterozygotes and non-carriers. In addition, baseline cerebral microhemorrhages, cortical superficial siderosis, and elevated mean arterial pressure are associated with an increased risk of ARIA (Zimmer et al., 2025). Current clinical practice recommendations increasingly advocate *APOE* genotyping and MRI-based risk assessment before anti-Aβ treatment to enable individualized risk stratification and safety monitoring. Although Lecanemab and Donanemab show robust amyloid-lowering effects and may theoretically reduce neurotoxic burden within sensory pathways, direct evidence for improvement in sensory function is still lacking. Future studies should incorporate olfactory identification tests, visual function assessments, retinal imaging biomarkers, and sensory network neuroimaging measures as clinical endpoints and include *APOE* genotype-based stratification to determine whether anti-Aβ therapies confer clinically meaningful benefits in sensory function.

#### 2.5.8 Non-pharmacological interventions

Non-pharmacological interventions might also offer therapeutic potential for alleviating sensory dysfunction in AD. Photobiomodulation reduces oxidative stress, neuroinflammation, and apoptosis while improving retinal function and cognitive performance in animal models of AD (Chen et al., 2025a; Gopalakrishnan et al., 2020). Sensory-based interventions, such as olfactory training, might indirectly modulate inflammatory states by enhancing neural plasticity (Hummel et al., 2023). Visual and olfactory processing depend on specific neural oscillatory dynamics, including gamma rhythms and theta–gamma coupling. These oscillatory patterns are disrupted in ageing and in models of AD and might be further impaired by inflammatory processes (Ahnaou et al., 2020b; Ahnaou et al., 2020a). Preliminary studies suggest that 40-Hz visual or auditory stimulation could confer benefits in persons with mild cognitive impairment or early AD (Ang et al., 2025). Proposed mechanisms include the reduced production of pro-inflammatory cytokines and enhanced microglial clearance of Aβ (Martorell et al., 2019). However, current evidence remains limited by small samples, heterogeneous stimulation protocols, and short follow-ups that underscore the need for larger, well-controlled clinical trials.

Collectively, targeting inflammatory signal amplification, amyloid pathology, and neural network dynamics might offer critical mechanistic entry points to treat sensory dysfunction in AD.

## 3 Discussion

Neuroinflammation has been implicated in the development and progression of sensory dysfunction in Alzheimer’s disease (AD) and may provide opportunities for earlier identification and intervention. This review summarizes the interactions between neuroinflammation and Aβ/tau pathology in the olfactory and visual-retinal systems and discusses potential therapeutic targets and intervention strategies within the receptor–intracellular signaling–inflammasome–effector framework. Current evidence supports an association between sensory dysfunction and AD-related pathology rather than a disease-specific relationship. Visual and olfactory impairment occurs in a variety of neurological and non-neurological disorders and should not be regarded as standalone diagnostic markers of AD. Nevertheless, sensory abnormalities frequently emerge during the preclinical and prodromal stages of AD and are associated with early pathological changes, including Aβ deposition, tau pathology, neuroinflammation, and oxidative stress (Woodward et al., 2017; Koronyo et al., 2023; Gaire et al., 2024; Yu et al., 2024). These features make sensory dysfunction relevant for mechanistic studies and potential clinical translation. Several limitations should be acknowledged. First, assessment tools and outcome measures for sensory dysfunction remain heterogeneous across studies. Second, most evidence for anti-inflammatory interventions is derived from animal models, and further optimization of safety, dosing, disease stage selection, and clinical endpoints is required for translation to clinical practice. Third, current studies mainly focus on single sensory modalities, whereas evidence regarding multisensory impairment and stage-specific interventions remains limited. Future studies should develop anti-inflammatory strategies targeting sensory systems and evaluate their effects on sensory and cognitive outcomes through prospective clinical investigations. Further research is also needed to clarify the relationships among sensory dysfunction, core AD pathology, biomarkers, and disease progression, and to determine its potential value in disease monitoring, risk stratification, and early intervention.
